# Role Stress and Psychological Distress Among Chinese Nurses During the COVID-19 Pandemic: A Moderated Mediation Model of Social Support and Burnout

**DOI:** 10.3389/fpsyt.2022.812929

**Published:** 2022-03-17

**Authors:** Yuting Xiao, Honghui Zhang, Qian Li, Shan Xiao, Ting Dai, Jia Guo, Yu Yu

**Affiliations:** ^1^Department of Nursing, Hunan Provincial People's Hospital, The First-Affiliated Hospital of Hunan Normal University, Hunan, China; ^2^Department of Hepatobiliary Diseases, Hunan Provincial People's Hospital, The First-Affiliated Hospital of Hunan Normal University, Hunan, China; ^3^Department of Clinical Nursing, Xiangya School of Nursing, Central South University, Hunan, China; ^4^Division of Prevention and Community Research, Department of Psychiatry, Yale School of Medicine, New Haven, CT, United States

**Keywords:** role stress, burnout, psychological distress, social support, mediator, moderator

## Abstract

**Objective:**

Nurses are at high risk of psychological distress including stress, depression, and anxiety due to low personnel density and high work demand. Despite mounting evidence showing that role stress is a risk factor for nurses' psychological distress, the mediating and moderating mechanisms underlying this relationship are less known. This study tests the mediation effect of burnout in the association between role stress and psychological distress, and whether this mediation is moderated by social support.

**Methods:**

A sample of 623 Chinese nurses were recruited from all hepatological surgery departments in Hunan Province and filled out an online questionnaire to collect data on socio-demographics, role stress, burnout, psychological distress, and social support. Mediation and moderation analyses were carried out in SPSS macro-PROCESS.

**Results:**

Burnout partially mediated the positive association between role stress and psychological distress. Social support moderated the indirect effect of role stress on psychological distress via burnout, with the effect being stronger for nurses with low social support than those with high social support.

**Conclusions:**

These findings demonstrated how role stress contributed to nurses' psychological distress both directly and indirectly through burnout, and how this indirect effect was moderated by social support. The results provide important practical implications for future prevention and intervention programs to improve nurses' mental health from multiple aspects such as decreasing role stress and burnout while increasing social support.

## Introduction

The Coronavirus disease 2019 (COVID-19) and its global pandemic have become a serious global mental health concern all over the world, bringing about a sharp increase in the number of mental health problems such as loneliness, depression, and anxiety (1, 2). The negative impact of the COVID-19 on mental health is especially predominant among healthcare providers who work in the front line to directly combat the virus (3–5). It has been widely reported that healthcare providers are faced with many challenges during the COVID-19 pandemic, including increased workloads and extended work hours, limited resources and support, lack of knowledge and preparedness to respond to the COVID-19, and fear of getting infected as well as infecting their families (6, 7). All these obstacles have significantly contributed to mental health problems among healthcare providers, leading to a high level of role stress, burnout, and psychological distress that need to be addressed urgently (6–8).

Globally, role stress is a common and widespread phenomenon in healthcare and nurses are particularly at high risk of role stress due to high job demands, rapidly changing environments, facing patient's suffering and death, work overload, and insufficient support, which has been highlighted in several literature reviews (9–12). This situation is even worsened by the widespread global shortage of nursing staff (13), which is estimated to reach 12.9 million by 2,035 based on the prediction of the ‘Third Global Forum on Human Resources for Health' by the World Health Organization (14). In developing countries with big and aging populations like China, nurses are under substantial role stress due to more severe nursing staff shortages. According to the most recent data from the National Health Commission of China, the total number of registered nurses is 4.70 million by the end of 2020, and the nurse density is 3.35 per thousand population (15). This number is far below the nurse density of 9–17 per thousand population in developed countries in Europe and North America (16).

Role stress is a leading contributor to psychological distress including stress, depression, and anxiety, which is also a prevalent phenomenon among nurses, especially under the global pandemic of Covid-19 (17, 18). A recent review and meta-analysis showed a pooled prevalence of 43 for stress, 37 for anxiety, and 35% for depression among nurses, indicating an alarmingly high level of psychological distress among nurses (19). Despite accumulating evidence demonstrating the positive effect of role stress on psychological distress among nurses, the mediating mechanism and moderating effect underlying this relationship, especially among Chinese nurses, are less known. The present study aims to validate the mediator of burnout and the moderator of social support between role stress and psychological distress among Chinese nurses.

The concept of role stress was first brought about by Kahn et al. (20, 21) in 1964 and has been defined as “anything about an organizational role that produces adverse consequences for the individual”. Role stress is characterized by three dimensions: role conflict, role ambiguity, and role overload. Role conflict occurs when a person faces incompatible demands between the expectations of two or more parties, or when a person feels contradiction between the external demands of his job and the internal standards of behavior in a single role (20–22). Role ambiguity refers to uncertainty about what actions to take for a job role and arises when a person has insufficient, vague, or unclear information to fulfill his job requirements adequately (20–22). Role overload refers to a person's lack of resources needed to fulfill commitments, obligations, or requirements, and happens when a person is faced with multiple obligations that require him to do more than he can handle at the time (20–22).

According to Schotte and Clum's diathesis-stress model (23), stress produces demands on individuals that require adaptation and resolution. Those who lack problem-solving skills may react poorly to stressors, which consequently lead to psychological distress such as stress, depression, and anxiety (23). From this perspective, numerous studies have consistently illustrated that a high level of role stress is associated with high levels of stress, depression, and anxiety (24–26).

Burnout is a psychological syndrome that develops in response to prolonged stressful working conditions and typically includes three dimensions: emotional exhaustion, depersonalization, and decreased personal accomplishment (27, 28). Emotional exhaustion is the first and key step of burnout and also a mandatory criterion for burnout syndrome, representing feelings of extreme tiredness and exhaustion emotionally due to excessive physical and emotional efforts (27, 28). Depersonalization occurs after emotional exhaustion and refers to the negative and callous attitudes toward patients, marked by cynicism, disinterest, and even dehumanization (27, 28). A high level of depersonalization may lead nurses to view their patients as deserving of their sufferings (28). The third aspect of burnout is decreased personal accomplishment, a tendency to evaluate oneself negatively, marked by unhappiness about oneself and dissatisfaction with one's job accomplishment (27, 28).

As a job-specific phenomenon, burnout has been a heated research topic in studies on nurses' well-being (29–31). Burnout is often seen as an outcome of role stress, with numerous studies demonstrating that nurses with higher levels of role stress are at higher risk of burnout (32–36). Additionally, abundant evidence also documented that burnout may lead to psychological distress such as stress, depression, and anxiety. For instance, Salvagioni et al. (37)conducted a systematic review of prospective studies on the physical, psychological and occupational consequences of job burnout. Their results showed that burnout significantly predicted a wide range of psychological disorders including insomnia, depressive symptoms, hospitalization for mental disorders, and psychological ill-health symptoms (37). Burnout may be the mechanism or the mediator through which role stress leads to psychological distress. In Khamisa et al.'s (38) systematic review of nurse burnout literature, they found nurse burnout mediated the relationship between work-related stressors and general health that includes psychological distress.

Although role stress may be related to psychological distress through burnout, not all nurses with role stress equally suffer from burnout and develop psychological distress. One of the most important and widely studied factors that may moderate the effect of role stress on psychological distress via burnout is social support. Social support is a multidimensional concept that involves various aspects of resources one perceived that come from various sources, such as family, friends, and co-workers (39, 40). An ever-growing number of studies and reviews have consistently documented the important protective role of social support on mental health and well-being (41–43). People with a higher level of social support have a lower level of psychological distress such as stress, depression, and anxiety (41–43). Social support can promote mental health both directly as a main effect, and indirectly as a moderating variable that buffers the effects of stress (39, 40). For instance, Etzion's (44) earlier study showed social support moderated the effect of the stress-burnout relationship, while the most recent evidence from Wang et al. (45) and Zhang et al.'s (46) study showed social support moderated the effect of job burnout on psychological distress.

While it has been well-established that higher role stress may lead to psychological distress, less well-known is the indirect effect of role stress on psychological distress via burnout, and whether this effect was moderated by social support. The Job Demand-Resources Model (J-DR model) provides a suitable model to understand the mediating and moderating mechanism underlying the relationship between role stress and psychological distress (47, 48). According to the J-DR model (47, 48), job characteristics include both a negative aspect named job demands (e.g., role stress) and a positive aspect named job resources (e.g., social support). High job demands may exaggerate the level of burnout and result in negative health outcomes, such as psychological distress (49–51). On the other hand, high job resources may foster nurses' engagement of work, self-accomplishment, and lead to better mental health, as well as buffer the negative effect of job demands on negative health outcomes (49–51).

**Figure 1:**
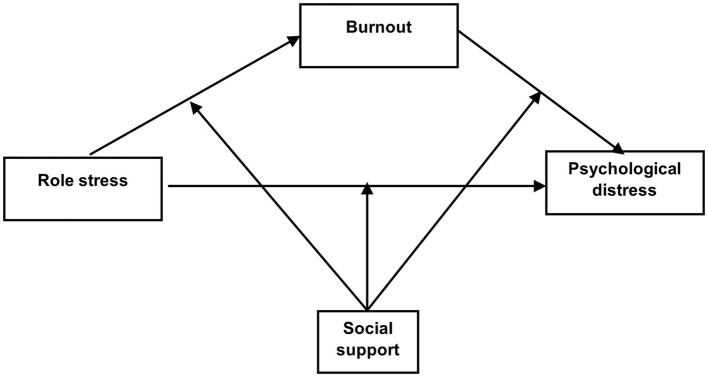
The proposed moderated mediation model.

Based on the J-DR model, we proposed this study's conceptual model as shown in Figure 1 to test a moderated mediation model of social support and burnout on the relationship between role stress and psychological distress among nurses. Specifically, we proposed the following hypotheses:

Hypothesis 1. Role stress would be positively associated with nurses' experience of psychological distress including stress, depression, and anxiety.
Hypothesis 2. Burnout would mediate the relationship between role stress and psychological distress.
Hypothesis 3. Social support would moderate the direct and indirect relations between role stress and psychological distress.

## Methods

### Participants and Procedure

This was a cross-sectional study conducted among nurses of hepatological surgery departments in Hunan Province from June to July 2021. For this study, the inclusion criteria consisted of direct care nurses working in hepatological surgery departments. We included all hospitals in Hunan Province that have set up an independent hepatological surgery department. We achieved information of all hepatological surgery departments in Hunan Province through the Hepatobiliary Health Education Chair of Hunan Province and contacted the head nurse of each department to get a list of nurses, leading to a sampling frame of 856 nurses in the hepatological surgery department. The study was conducted online, with all questionnaires produced, distributed, and collected with the online survey tool Sojum (52). Sojum is a professional online survey tool that provides a series of services including questionnaire design and distribution, data collection and analysis. We sent the questionnaire link to each hepatological surgery department head nurse, who then distributed the link to all nurses in her department. Among the 856 nurses invited to the online survey, 146 refused to respond. Finally, we collected 623 valid questionnaires after eliminating 38 questionnaires with obvious logical mistakes and 49 questionnaires with a too short response time (3 SD below the average response time). Ethics approval was obtained from the Institutional review board (IRB) of the Hunan Provincial People's Hospital (No. Ky-2021-42). All participants provided written informed consent before participating in the study and finished the online questionnaires on their own.

Six hundred and twenty-three nurses completed the questionnaire. All nurses are required to fill out each item of the questionnaire to be able to submit the online questionnaire and as a result, there is no missing data in the final dataset. Most of the nurses are female (98.72%), and only 8 (1.28%) were male. The nurses' age ranges from 20 to 58, with an average age of 30.88 (SD = 6.14). Two-thirds of nurses were married (66.93%), and most had an education level of college and above (81.86%). Their working years in nursing ranges from 0.5 to 37, with an average year of 9.48 ± 6.52. In terms of professional and technical titles, the primary level accounted for 50.9%, the middle level accounted for 42.4%, while the higher level accounted for 6.7%.

### Measures

#### Role Stress

Role stress was measured using the Role Conflict, Ambiguity and Overload Scale (RCAOS) developed by Peterson (22). The RCAOS was first translated into Chinese by Li and Zhang (53) and has been well-validated among Chinese populations to measure various aspects of role stress (53, 54). The RCAOS includes 13 items under three subscales: role conflict (three items), role ambiguity (five items), and role overload (five items) (22). Each item is rated on a five-point Likert scale ranging from “1” (very opposed) to “5” (very much agree). All five items of the role ambiguity subscale were reversely coded. The total score of the RCAOS ranges from 13–65, with higher scores indicating higher levels of role stress. The RCAOS has demonstrated favorable psychometric properties in previous studies (22, 34, 53, 54). In the current study, the RCAOS showed good internal consistency with a Cronbach's alpha coefficient of 0.83.

#### Burnout

Burnout was measured using the Maslach Burnout Inventory (MBI) developed by Maslach and Jackson (28). The MBI is the most widely used well-established instrument for measuring burnout at the workplace all over the world, with favorable reliability and validity reported in various samples across cultures and countries (55–57). The MBI includes 22 items under three subscales: emotional exhaustion (EE) (9 items), depersonalization (DP) (5 items), and personal accomplishment (PA) (8 items) (28). Each item is rated on a seven-point Likert scale ranging from ‘0' (never) to “six” (every day). For easy combination, we reverse-coded each item of the PA to get a transformed subscale— decreased personal accomplishment (DPA) (34). The total score of the MBI ranges from 0–132, with higher scores indicating higher levels of burnout. The MBI has demonstrated good psychometric properties in previous studies (55–57). In the current study, the MBI showed good internal consistency with a Cronbach's alpha coefficient of 0.91.

#### Psychological Distress

Psychological distress was measured using the Depression Anxiety Stress Scale 21 (DASS-21) developed by Lovibond and Lovibond (58). The DASS-21 is the most widely used and well-established instrument for measuring psychological distress all over the world, with favorable reliability and validity reported in both clinical and non-clinical samples across cultures and countries (59–61). The DASS-21 includes three 7-item subscales: depression, anxiety, and stress (58). Each item is rated on a four-point Likert scale ranging from “0” (does not apply to me) to “3” (applies to me most of the time). The total score of the DASS-21 ranges from 0–63, with higher scores representing higher levels of psychological distress. The DASS-21 has demonstrated good psychometric properties in previous studies (59–61). In the current study, the DASS-21 showed excellent internal consistency with a Cronbach's alpha coefficient of 0.96.

#### Social Support

Social support was measured using a self-designed scale that included three questions asking about the level of support participants received from family, supervisor, and colleagues. Each item is rated on a five-point Likert scale ranging from “1” (extremely unsupportive) to “5” (extremely supportive). Sample items included “To what degree is your family supportive of your nursing work?”. The total score ranges from 3 to 15, with higher scores representing higher levels of social support. In the current study, the social support scale showed acceptable internal consistency with a Cronbach's alpha coefficient of 0.70.

### Data Analyses

All data analyses were conducted using SPSS v 23.0. First, descriptive analysis and correlation analysis were conducted among variables. Second, a simple mediation analysis of burnout mediating the relationship between role stress and psychological distress was tested using Hayes's PROCESS macro for SPSS (Model 4) (62). Third, a moderated mediation analysis was conducted using Hayes' PROCESS macro (Model 59) to test the moderating role of social support in all possible direct and indirect effects of role stress on burnout in the mediation model (62). The indirect effect of mediation was tested using a bootstrapping method with 5,000 samples as recommended, with a significant effect indicated by a 95% confidence interval not including zero (62). All analyses were controlled for nurses' socio-demographics including age, gender, marital status, education, working years, and professional and technical titles.

## Results

### Descriptive Statistics and Correlation Analysis

Table 1 presents the means and SDs and the Pearson correlations of study variables. The mean and SD of each study variable was 36.13 ± 6.92 for role stress, 66.30 ± 20.65 for burnout, 14.83 ± 11.63 for psychological distress, and 12.39 ± 1.70 for social support. Pearson correlations analyses showed that role stress was positively associated with burnout (*r* = 0.39, *p* < 0.001) and psychological distress (*r* = 0.50, *p* < 0.001), but negatively associated with social support (*r* = −0.42, *p* < 0.001). Burnout was positively associated with psychological distress (*r* = 0.51, *p* < 0.001), but negatively associated with social support (*r* = −0.26, *p* < 0.001). Psychological distress was negatively associated with social support (*r* = −0.36, *p* < 0.001).

**Table 1:** Descriptive statistics and correlations among variables.

	**M ±SD**	**1**	**2**	**3**	**4**
1. Role stress	36.13 ± 6.92	1			
2. Job burnout	66.30 ± 20.65	0.39^***^	1		
3. Psychological distress	14.83 ± 11.63	0.50^***^	0.51^***^	1	
4. Social support	12.39 ± 1.70	−0.42^***^	−0.26^***^	−0.36^***^	1

*** *p < 0.001 (2-tailed)*.

### Testing for Mediating Effect

Table 2 shows the results of mediating effect of burnout. Role stress had a significant positive effect on psychological distress (β = 0.84, *t* = 14.30, *p* < 0.001), and on burnout (β = 1.15, *t* = 10.34, *p* < 0.001). When the mediating variable burnout was added, the direct effect of role stress on psychological distress was still significant (β = 0.59, *t* = 10.20, *p* < 0.001), indicating partial meditation. Burnout had a significant positive effect on psychological distress (β = 0.21, *t* = 11.00, *p* < 0.001).

**Table 2:** Testing the mediation effect of role stress on psychological distress through job burnout^a^.

**Predictors**	**Model 1(Psychological distress)**			**Model 2 (Job burnout)**			**Model 3 (Psychological distress)**		
	**B**	**SE**	***p***	**B**	**SE**	***p***	**B**	**SE**	***p***
Role stress	0.84	0.06	<0.001	1.15	0.11	<0.001	0.59	0.06	<0.001
Job burnout							0.21	0.02	<0.001
*R* ^2^	0.26			0.16			0.38		
*F*	30.30^***^			16.64^***^			46.81^***^		

*Each column is a regression on model that predicts the criterion at the top of the column*.

*^a^All socio-demographic variables (age, working years, gender, marital status, education, and professional and technical titles) were controlled as covariates, and nonsignificant, thus not listed in the table*.

*** *p < 0.001*.

Further bootstrapping results showed the total effect of role stress on psychological distress was 0.84 (95% CI: 0.72, 0.96), with a direct effect of 0.59 (95%CI: 0.48, 0.71), and an indirect effect of 0.25 (95% CI: 0.18, 0.32) through burnout. All these intervals didn't include zero, indicating that burnout partially mediated the relationship between role stress and psychological distress, with an indirect effect accounting for 29.76% of the total effect.

### Testing for Moderated Mediation

In our proposed moderation mediation model, we hypothesized that social support would moderate all pathways in the mediation process of role stress on psychological distress through burnout. Table 3 shows the results of such moderation mediation analysis using Model 59 of PROCESS macro by Hayes (62). After social support was put into the model, role stress had a significant positive effect on psychological distress (β = 0.50, *t* = 7.77, *p* < 0.001), and the product of role stress and social support had no significant effect on psychological distress (β = 0.01, *t* = 0.39, *p* = 0.70), indicating that social support didn't moderate the direct effect of role stress on psychological distress.

**Table 3:** Testing the moderated mediation effect of social support on the relation between role stress and psychological distress via job burnout.

**Predictors**	**Model 1 (Psychological distress)**			**Model 2 (Job burnout)**			**Model 3 (Psychological distress)**		
	**B**	**SE**	***p***	**B**	**SE**	***p***	**B**	**SE**	***p***
Role stress	0.74	0.07	<0.001	1.08	0.13	<0.001	0.50	0.06	<0.001
Social support	−1.11	0.27	<0.001	−1.16	0.51	0.023	−0.90	0.25	<0.001
Role stress X social support	−0.05	0.04	0.15	−0.16	0.07	0.02	0.01	0.03	0.696
Job burnout							0.20	0.02	<0.001
Job burnout X Social support							−0.03	0.01	0.008
*R* ^2^	0.28			0.18			0.40		
*F*	26.98^***^			14.75^***^			37.03^***^		

*Each column is a regression model that predicts the criterion at the top of the column*.

*** *p < 0.001*.

**Figure 2:**
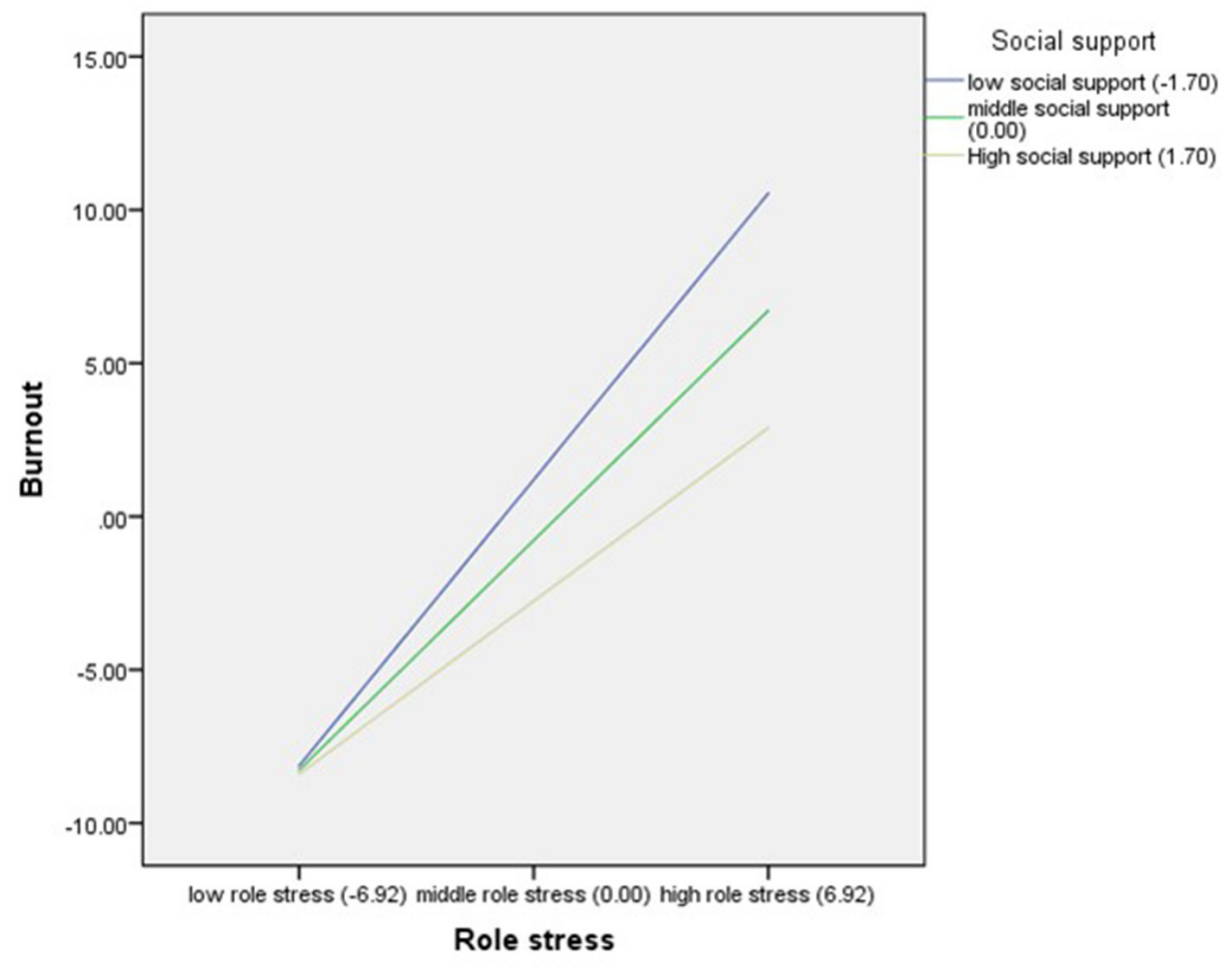
Social support as a moderator between role stress and burnout.

Role stress had a significant positive effect on burnout (β = 1.08, *t* = 8.54, *p* < 0.001), and the product of role stress and social support had a significant effect on burnout (β = −0.16, *t* = - 2.34, *p* = 0.02), indicating that social support moderated the effect of role stress on burnout. Further simple slope analysis results are shown in Figure 2. For participants with low social support level (M-1SD), role stress had a significant positive effect on burnout (*b*_*simple*_ = 1.35, *t* = 6.97, *p* < 0.001). As the social support increased to the middle (M) and high level (M + 1SD), role stress also had a positive effect on burnout, but the effect was decreasing gradually (middle level: *b*_*simple*_ = 1.08, *t* = 8.54, *p* < 0.001; high level: *b*_*simple*_ = 0.82, *t* = 5.68, *p* < 0.001), indicating that with the improvement of social support level, the positive effect of role stress on burnout was decreasing gradually.

**Figure 3:**
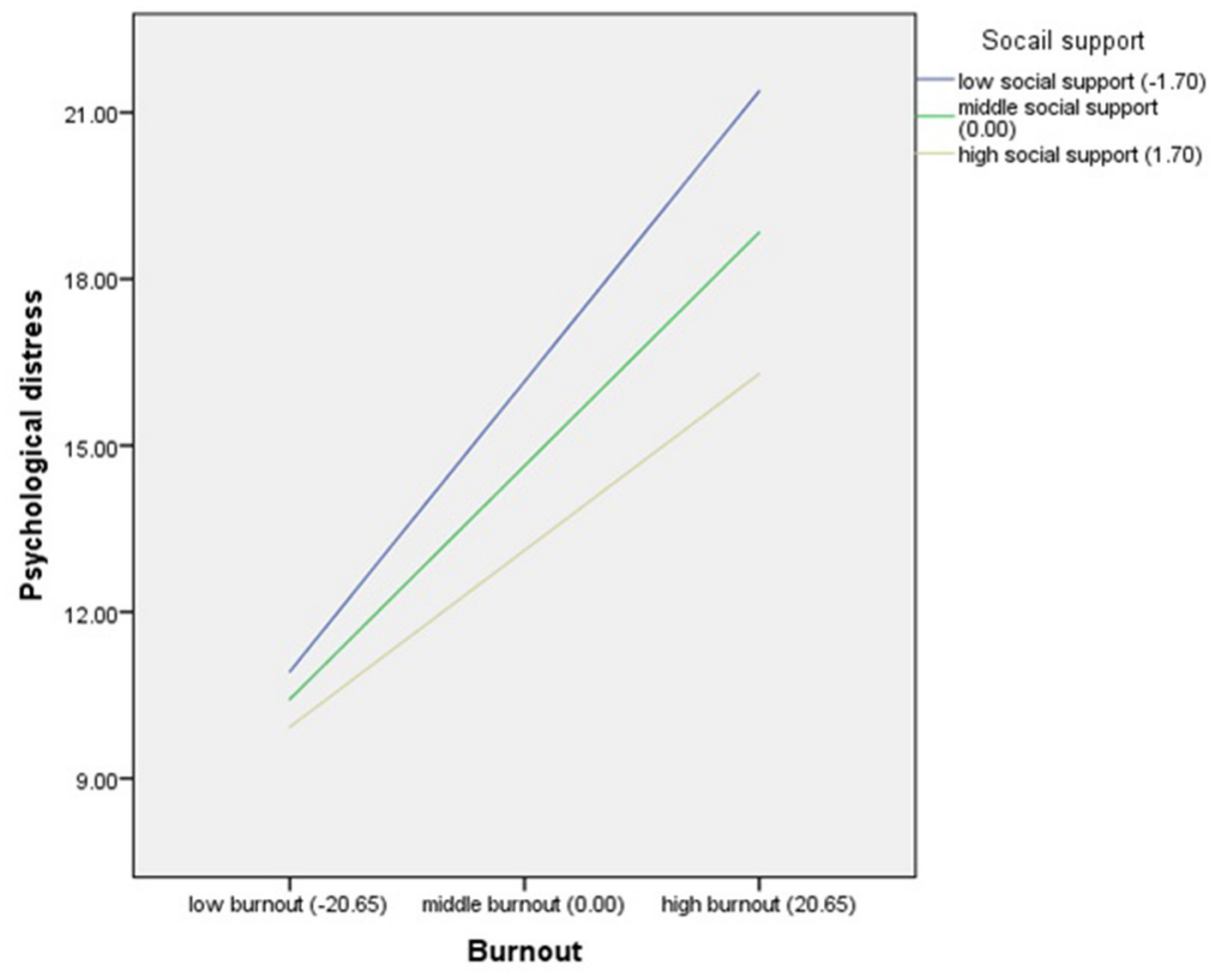
Social support as a moderator between burnout and psychological distress.

Burnout had a significant positive effect on psychological distress (β = 0.20, *t* = 10.46, *p* < 0.001), and the product of burnout and social support had significant effect on psychological distress (β = −0.03, *t* =-2.66, *p* = 0.008), indicating that social support moderated the effect of burnout on psychological distress. Further simple slope analysis results are shown in Figure 3. For participants with low social support level (M-1SD), burnout had a significant positive effect on psychological distress (*b*_*simple*_ = 0.25, *t* = 9.55, *p* < 0.001). As the social support increased to the middle (M) and high level (M + 1SD), burnout also had a positive effect on psychological distress, but the effect was decreasing gradually (middle level: *b*_*simple*_ = 0.20, *t* = 10.46, *p* < 0.001; high level: *b*_*simple*_ = 0.15, *t* = 5.64, *p* < 0.001), indicating that with the improvement of social support level, the positive effect of burnout on psychological distress was decreasing gradually.

## Discussion

This study explored how role stress affected psychological distress among nurses using a moderated mediation model. First, as expected, burnout mediated the relationship between role stress and psychological distress, supporting our hypotheses 1 & 2. Second, social support moderated the indirect effect of role stress on psychological distress through burnout, but not the direct effect of role stress on psychological distress, partially supporting our hypothesis 3. The results deepen our understanding of the mechanism underlying role stress and psychological distress and identify important factors that may enhance or decrease the strength of such an association.

### The Mediating Role of Job Burnout

In line with the diathesis-stress model (23), our study showed that role stress was positively associated with nurses' psychological distress, with or without controlling for job burnout. This result is consistent with previous studies showing role stress leading to psychological distress and provides new evidence for the diathesis-stress model (24–26). Role stress includes three dimensions: role conflict, role ambiguity, and role overload. The impact of role conflict and role ambiguity is pervasive and most widely studied. When nurses receive insufficient or contradictory information on what actions to take to perform a task, they may feel a high level of job tensions and a low level of job satisfaction, leading to psychological distress such as stress, depression, and anxiety (24, 63). Role overload, although less frequently studied, also plays a significant role in contributing to psychological distress. Nurses that are overloaded with more work than they can handle with are more likely to suffer from psychological distress than nurses who are assigned with appropriate workloads (64, 65).

Our results indicated that burnout mediated the relationship between role stress and psychological distress, which is also in accordance with the large body of literature showing the mediating role of burnout in work stress and psychological distress relations (38). For instance, both Chen et al.'s (43) and Zhang et al. (66) studies demonstrated that emotional exhaustion, a key aspect of burnout, mediated the relationship between job stress and anxiety. These findings further enrich our knowledge on the mediating mechanisms to explain the association between role stress and psychological distress among Chinese nurses. Aligning with the J-DR model (47, 48), burnout mediated the relationship between role stress and psychological distress. When nurses are confronted with role conflict, role ambiguity, or role overload, they may feel they cannot fulfill their job requirements. In the long run, nurses with high role stress may experience emotional exhaustion, followed by depersonalization, and decreased personal accomplishment, and finally resulting in psychological distress such as stress, depression, and anxiety.

### The Moderating Role of Social Support

Drawing on the J-DR model (47, 48), we found social support moderated the indirect effect of role stress on psychological distress via burnout. Our results highlighted the buffering role of social support in affecting psychological distress through burnout among Chinese nurses. These results agreed with most of the previous research demonstrating the moderating effect of social support in the association between role stress and burnout (44), as well as in the association between burnout and psychological distress (45, 46). Moreover, Zhang et al. (66) reported that social supported moderated the relationship between work-family conflict and anxiety symptoms through emotional exhaustion, a key aspect of burnout. Our study built on their findings and provided a more comprehensive investigation on the relationship between role stress and psychological distress via burnout. Based on the J-DR model, job resources (e.g., social support) play a buffering effect on the effect of job demands (e.g., role stress) on psychological distress through burnout. Compared to nurses with a lower level of social support, nurses with a higher level of social support may experience less obvious effect of role stress on psychological distress through burnout.

### Limitations and Practical Implications

Although the present study advances our understanding of the mediating and moderating mechanism underlying the association between roles stress and psychological distress among Chinese nurses, several limitations need to be acknowledged. First, our sample was recruited from all hepatological surgery departments in Hunan Province, which may not represent nurses in other departments, or in other provinces. However, our large sample size using a whole sampling method from all hepatological surgery departments in Hunan Province still provides a relatively unbiased sample to help us understand role stress and psychological distress in hepatological nurses. In the future, it may be worthwhile to conduct national-level, multi-center studies to get a more comprehensive overview of nurses' well-being in China. Second, the cross-sectional study design may preclude any causal relationship conclusions to be drawn from the study. However, this study was built on solid theoretical and empirical evidence, we believe our findings still provide useful and important information on the moderated mediation model between role stress and psychological distress. Future longitudinal study designs are needed to validate the causal relationship more robustly in this model. Third, social support was measured using self-designed three-item questionnaires instead of standard social support scales, which may make it difficult to make comparisons with other studies using standard scales. However, our three-item questionnaires directly tapped three sources of support: family, supervisor, and colleagues that are closely and directly associated with nurses' work-related stress. Besides, the questionnaires showed acceptable internal consistency with a Cronbach alpha of 0.70, indicating the reliability of the questionnaire in measuring social support. Future studies may consider using a standard social support scale to better capture social support and ensure cross-study comparisons. Fourth, all study variables were measured using self-report questionnaires, which may lead to potential bias and affect the accuracy of the assessment. Future studies may consider using more objective measurement tools to accurately capture these concepts.

Despite these limitations, our findings still carry significant practical implications. First, consistent with other research, our findings suggest that role stress is a significant risk factor for psychological distress. This result indicates that future research and intervention targeting alleviating nurses' psychological distress should start with decreasing their roles stress level, which may be realized through clear and consistent instructions of role tasks, and lessened workload. Second, given that burnout is a significant mediator linking role stress and psychological distress, reducing the level of burnout among nurses may be another helpful and efficient measure to reduce and prevent psychological distress. For instance, psycho-social interventions aimed at alleviating the feelings of emotional exhaustion, decreasing the level of depersonalization, and improving self-achievement may best serve the purpose of improving and preventing nurses' psychological distress. Third, this study has demonstrated the buffering role of social support in the indirect relationship between role stress and psychological distress among nurses. Considering the moderating role of social support in both the relationship between role stress and burnout, as well as the relationship between burnout and psychological distress, improving the level of social support that nurses receive may also greatly help decrease psychological distress. For example, family members may share more family responsibilities such as doing housework and caring for kids to relieve nurses from the burden of family caregiving to avoid any family-work conflict that may increase role stress. Supervisors should be more caring and understanding, while colleagues may be more collaborative and cooperative, to create a more supportive working environment for the nurses. Finally, our study was conducted during the COVID-19 pandemic and the findings may provide useful guidance for improving mental health among all health care providers not only in China but also in other countries that have been affected by the COVID-19 pandemic. As evidence has shown that mental health support is urgently needed for health care providers all over the world in combating the COVID-19 pandemic to relieve their psychological distress (3, 6, 8). Our studies suggest that decreasing role stress and burnout, as well as strengthening social support among healthcare providers may be an effective and useful way to improve their mental health under the COVID-19 pandemic. These strategies may also be generalized to a broader public that suffers from covid pandemic as well.

## Conclusions

Although much research attention has been directed toward understanding the effect of role stress on psychological distress, less attention has been paid to the mediation and moderation mechanisms underlying such an association among nurses. This study suggests that role stress was associated with psychological distress both directly and indirectly through burnout, the latter was further moderated by social support. A higher level of social support may buffer the indirect effect of role stress on psychological distress. The findings provide important practical implications for future intervention and prevention programs targeted at alleviating nurses' psychological distress, which may be realized through reducing the level of role stress and burnout, while also strengthening the level of social support.

## Data Availability Statement

The raw data supporting the conclusions of this article will be made available by the authors, without undue reservation.

## Ethics Statement

The studies involving human participants were reviewed and approved by the Institutional Review Board (IRB) of the Hunan Provincial People's Hospital (No. Ky-2021-42). The patients/participants provided their written informed consent to participate in this study.

## Author Contributions

YX, HZ, and YY contributed to the conception and design of the study. YX, HZ, QL, SX, TD, and JG contributed to the research conduction and data collection. YX, HZ, and QL contributed to data analyses, while SX, TD, JG, and YY contributed to data interpretation. YX drafted the article while HZ, QL, SX, TD, JG, and YY critically appraised and revised it. All authors approved the final version of the manuscript for submission.

## Funding

This work was supported by the Hunan Provincial Science and Technology Department (No. 2020ZK4063), the Education Department of Hunan Province (No. 20C1121), and the Health Commission of Hunan Province (No. 202114021956) in support of the corresponding author. The funder had no role in the design of the study, collection, analysis, interpretation of data, and in writing this manuscript.

## Conflict of Interest

The authors declare that the research was conducted in the absence of any commercial or financial relationships that could be construed as a potential conflict of interest.

## Publisher's Note

All claims expressed in this article are solely those of the authors and do not necessarily represent those of their affiliated organizations, or those of the publisher, the editors and the reviewers. Any product that may be evaluated in this article, or claim that may be made by its manufacturer, is not guaranteed or endorsed by the publisher.
